# A Dual Ligand Sol–Gel Organic-Silica Hybrid Monolithic Capillary for In-Tube SPME-MS/MS to Determine Amino Acids in Plasma Samples

**DOI:** 10.3390/molecules24091658

**Published:** 2019-04-27

**Authors:** Luis F. C. Miranda, Rogéria R. Gonçalves, Maria E. C. Queiroz

**Affiliations:** Departamento de Química, Faculdade de Filosofia Ciência e Letras de Ribeirão Preto, Universidade de São Paulo, P.O. Box 14040-901 Ribeirão Preto, SP, Brazil; luisfelipe.c22@usp.br (L.F.C.M.); rrgoncalves@ffclrp.usp.br (R.R.G.)

**Keywords:** in-tube SPME-MS/MS, dual ligand organic-silica hybrid monolith capillary, amino acids, plasma samples

## Abstract

This work describes the direct coupling of the in-tube solid-phase microextraction (in-tube SPME) technique to a tandem mass spectrometry system (MS/MS) to determine amino acids (AA) and neurotransmitters (NT) (alanine, serine, isoleucine, leucine, aspartic acid, glutamic acid, lysine, methionine, tyrosine, and tryptophan) in plasma samples from schizophrenic patients. An innovative organic-silica hybrid monolithic capillary with bifunctional groups (amino and cyano) was developed and evaluated as an extraction device for in-tube SPME. The morphological and structural aspects of the monolithic phase were evaluated by scanning electron microscopy (SEM), Fourier transform infrared spectroscopy (FTIR), nitrogen sorption experiments, X-ray diffraction (XRD) analyses, and adsorption experiments. In-tube SPME-MS/MS conditions were established to remove matrix, enrich analytes (monolithic capillary) and improve the sensitivity of the MS/MS system. The proposed method was linear from 45 to 360 ng mL^−1^ for alanine, from 15 to 300 ng mL^−1^ for leucine and isoleucine, from 12 to 102 ng mL^−1^ for methionine, from 10 to 102 ng mL^−1^ for tyrosine, from 9 to 96 ng mL^−1^ for tryptophan, from 12 to 210 ng mL^−1^ for serine, from 12 to 90 ng mL^−1^ for glutamic acid, from 12 to 102 ng mL^−1^ for lysine, and from 6 to 36 ng mL^−1^ for aspartic acid. The precision of intra-assays and inter-assays presented CV values ranged from 1.6% to 14.0%. The accuracy of intra-assays and inter-assays presented RSE values from −11.0% to 13.8%, with the exception of the lower limit of quantification (LLOQ) values. The in-tube SPME-MS/MS method was successfully applied to determine the target AA and NT in plasma samples from schizophrenic patients.

## 1. Introduction

Schizophrenia is a syndrome of inconclusive etiopathogenesis with a prevalence of about 1% in the general population. Underlying factors include genetic predisposition and impaired neurodevelopment in early life stages [1]. 

In the past few years, interest in finding out a possible role of amino acids (AA) in schizophrenia pathophysiology has increased [2,3,4,5,6,7,8,9]. Higher glycine, serine, glutamate, and aspartic acid concentrations were reported in plasma samples from schizophrenic patients [2,10]. Levels of other AA have provided inconsistent results [4]. In this context, many researchers have monitored AA and neurotransmitters (NT) in schizophrenic patients [2,6,7,9].

Liquid chromatography tandem–mass spectrometry (LC-MS/MS) has been used to determine AA and NT concentrations in biological samples from schizophrenic patients [2,9,11]. Sample preparation is a significant step in any bioanalytical chromatographic procedure even when powerful analytical instruments are employed.

In-tube solid-phase microextraction (in-tube SPME) is an effective sample preparation technique for biological fluids. It is fast to operate, easy to automate, solvent-free, and requires small sample volume. Capillaries with different stationary phases, including monolithic polymers, have been used in the first dimension of the in-tube SPME-LC system [12,13,14,15,16,17,18,19,20,21,22,23,24,25]. Monolithic materials have binary porous structure (mesopores and macropores). The presence of micron-size macropores ensures fast dynamic transport and low backpressure, leading to high flow rate and analytical speed. Moreover, polymeric monolith presents satisfactory loading capacity (which is superior to open tubular column loading capacity) and favors a convective mass transfer procedure (which is preferable in extraction processes). Organic-inorganic hybrid silica-based monoliths combine the advantages of organic polymers (pH stability and good biocompatibility) and silica-based monoliths (high permeability, high mechanical strength, and good organic solvent tolerance) [26,27,28,29,30].

Organic-inorganic hybrid silica-based monolithic capillaries with cyano [31,32,33] or amino functionalities [34] have been developed as extraction device for microextraction. To our knowledge, preparation of an organic-inorganic hybrid silica-based monolithic capillary with both of these bifunctional groups (cyano and amino) is an innovation.

Newly developed methods in the mass spectrometry field include direct coupling of sample preparation devices, such as solid-phase microextraction (SPME), to the MS instrumentation [35,36]. 

In this context, the present article describes direct coupling of the in-tube SPME technique to the MS/MS system to determine alanine, serine, leucine, isoleucine, tryptophan, methionine, tyrosine, lysine, aspartic acid, and glutamic acid in plasma samples from schizophrenic patients. This system employs an organic-inorganic hybrid silica-based monolithic capillary bearing bifunctional groups (amino and cyano).

## 2. Results

### 2.1. Hybrid Monolithic Capillary Synthesis

Fused silica capillary pretreatment was important to clean and increase the concentration of silanol groups in the inner surface; these groups can act as chemical binding sites for effective monolith attachment during in situ sol–gel synthesis [37].

In general, the sol–gel reaction encompasses three steps: (a) alkoxysilane precursor hydrolysis; (b) condensation between hydrated silica (Si-OH groups) and non-hydrolyzed alkoxysilane to form siloxane bonds (Si–O–Si); and (c) polycondensation of additional silanol group linkage to form linear or cyclic oligomers and, eventually, a silicate network [38]. The sol–gel matrix properties such as pore size and sorption capacity can be controlled by changing monomer amount and type, water amount, pH, solvent nature, additives, and reaction temperature. 

APTES (3-aminopropyl triethoxysilane) is a basic alkoxide precursor that can promote fast TEOS (Tetraethylorthosilicate) hydrolysis (due to the hydroxyl group) and condensation [34,39]. On the other hand, the reaction between CN-TEOS (3-cyanopropyltriethoxysilane) and TEOS is slower in basic medium. Therefore, the main challenge of this synthesis is to elevate CN-TEOS hydrolysis and condensation reaction rates in basic medium to obtain a dual-ligand (cyane and amino) sol–gel organic-inorganic hybrid monolith. To achieve this goal, we used ammonium fluoride as catalyst. Fluoride can increase silicon coordination above four due to the smaller ionic radius of the fluoride anion as compared to the hydroxyl group [40,41,42]. Thus, ammonium fluoride promotes simultaneous hydrolysis and condensation of both precursors (CN-TEOS and APTES) with TEOS. 

Malik et al. have proven that the cyanopropyl moiety in CN-PDMS coatings provides effective extraction of highly and medium polar analytes from aqueous medium [43]; Yan et al. described that amino groups (hybrid silica monoliths) interact with acidic analytes [39]. Moreover, the precursors CN-TEOS and APTES can establish dipole–dipole, dipole–induced dipole, and charge–transfer interactions [31,43].

The optimization of surfactant and water amounts could help to control pore size and, consequently, permeability [39]. The CTAB surfactant acts as supramolecular template during sol–gel monolith formation and can be easily removed by simple solvent extraction. However, in our experiments, the CTAB amount was slightly different (5 and 7 mg), so it did not significantly influence analyte sorption. 

**Figure 1:**
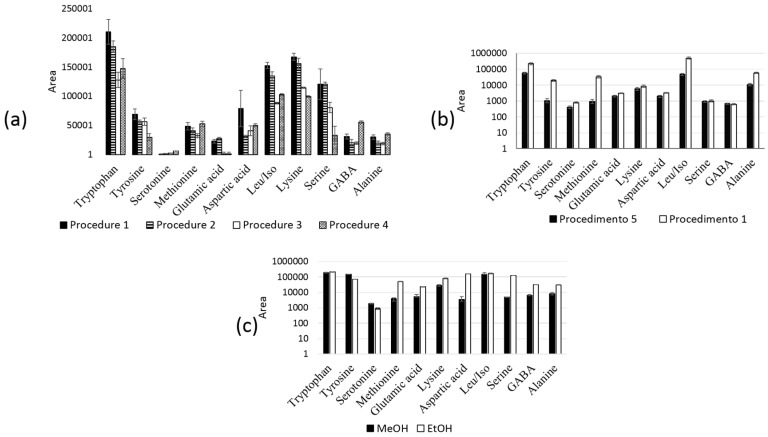
Optimization of the synthesis procedure (Table 1); (**a**) Molar ratios of the precursors TEOS/APTES/CN-TEOS, procedure 1 (2:1:1 *v*/*v*/*v*); procedure 2 (1:1:1 *v*/*v*/*v*); procedure 3 (1:1:0 *v*/*v*/*v*); and procedure 4 (1:0:1 *v*/*v*/*v*); (**b**) ethanol/water ratios [procedure 1 (107:20 μL) and procedure 5 (50:50 μL)]; (**c**) methanol or ethanol as solvent. Leu = leucine; Iso = isoleucine; GABA = γ-aminobutiric acid.

Initially, we investigated different molar ratios of the precursors (TEOS, CN-TEOS, and APTES) (Table 1, procedures 1−4). According to Figure 1a, procedure number 1 presented the highest signal area for majority of the analytes. Thus, the presence of both amino and cyano groups in the monolith structure increased the capillary sorption capacity. All the evaluated synthesis procedures resulted in monolithic phase with adequate permeability and high mechanical strength. The ethanol/water ratio (100:20 *v*/*v*) used in procedure number 1 improved capillary performance (Figure 1b). Methanol was also evaluated as a replacement for ethanol, but this change did not modify the sorption capacity (Figure 1c). The aging temperature influence was assessed in procedure number 6 (Table 1). The monolithic phase prepared at 22 °C was not reproducible—the correlation coefficient was higher than 15%. Hence, we selected 60 °C as the aging temperature for subsequent assays.

Three new different capillaries synthesized by procedure 1 attested that the in-situ polymerization procedure was reproducible. We assayed these capillaries with 100 nmol mL^−1^ AA and NT aqueous solution. The intra-batch and inter-batch assays presented RSD values lower than 15.0%, which demonstrated that the synthesis procedure had good reproducibility.

### 2.2. Characterization of Hybrid Silica Monoliths

**Figure 2:**
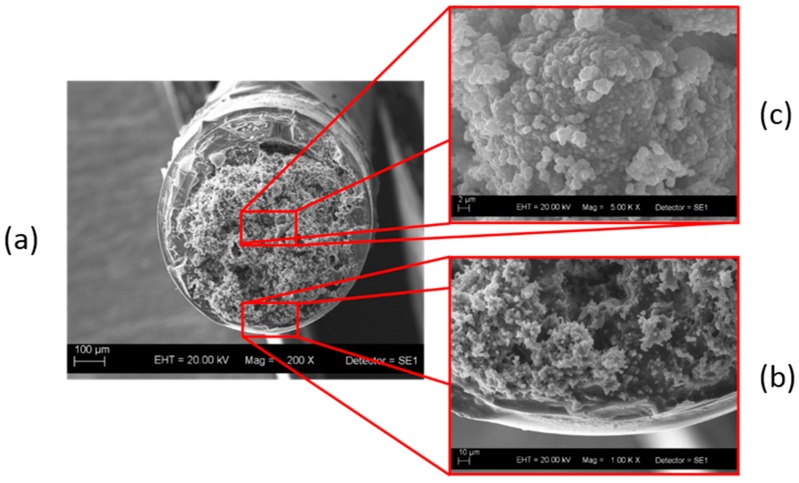
Scanning electron microscopy images of hybrid silica monolith containing cyanopropyl and aminopropyl groups. (**a**) Magnification of 200 X; (**b**) magnification of 1.00 kX; (**c**) magnification of 5.00 kX.

The SEM micrographs in Figure 2 show the morphological features of the monolithic capillary. Because we performed the reaction in the presence of ammonium fluoride as basic catalyst, we expected that the morphological features of the hybrid system would resemble the morphological features of the material obtained from TEOS in the presence of the APTES amino groups (basic precursor).

According to Figure 2, the hybrid monolithic capillary did not present any shrinkage and was uniform and regular. The monolith was clearly tightly attached to the capillary inner wall (Figure 2b). Both SEM images evidenced a homogeneous, continuous, and porous skeleton consisting of interconnected particles. The faster condensation kinetics during the ammonium fluoride-catalyzed sol–gel processes generated a highly compacted particulate structure. Morphological features are extremely important to understand microstructure and pore distribution as well as their correlation with sorption efficiency. Nitrogen sorption experiments offered a deeper understanding of porosity: the specific BET surface area and pore volume were 64.12 m^2^ g^−1^ and 0.064 cm^3^ g^−1^, respectively. Compared to the cyanoethyl monolithic sorbent reported by Souza et al. [32], the monolithic sorbent synthesized here had smaller pore volume and larger surface area.

**Figure 3:**
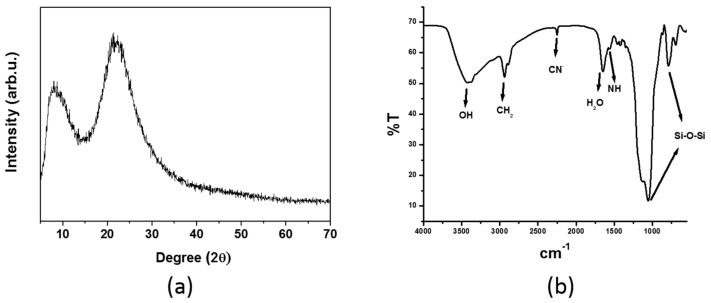
(**a**) XRD of hybrid silica monolith containing cyanopropyl and aminopropyl groups. (**b**) FTIR spectrum of hybrid silica monolith containing cyanopropyl and aminopropyl groups.

Figure 3a illustrates the XRD pattern of the chemically modified silica. There was a broad band in the 2θ region between 15° and 40°, with maximum at 22°, which corresponded to the amorphous silica-based host. The absence of a peak at higher angles confirmed that the silica was amorphous. However, the diffraction peak at 8.2° suggested that the chemically modified silica had mesoporous structure, which resulted from the use of the CTAB surfactant, as pore template, and functionalized monomers (CN-TEOS and APTES) [44]. 

Figure 3b contains the FTIR absorption spectrum of the hybrid silica monolith functionalized with cyano and amino groups. The peaks at 2950 and 2254 cm^−1^ referred to C–H stretching and –CN stretching vibrational modes, respectively, and corroborated the presence of cyanopropyl groups in the silica network. The bands at 1435 and 1655 cm^−1^ were attributed to C–H bending vibrational modes and molecular water scissor bending vibration, respectively. A broad band at about 3500 cm^−1^ also evidenced the presence of water molecule and silanol groups and was assigned to O–H stretching vibrational modes and significant hydrogen bonding. The shoulder at 1558 cm^−1^ and the overlapped bands at about 3300–3400 cm^−1^ corresponded to NH_2_ vibrational modes and attested that amino groups were incorporated into the hybrid silica monolith. The stretching band at 795 cm^−1^ indicated that Si–C bonds existed in the prepared hybrid silica monolith. The bands located at 800 and 1100 cm^−1^ were ascribed to Si–O symmetric stretching and to Si–O–Si anti-symmetric stretching, respectively [45,46,47,48,49,50], which are typical of a silica network.

### 2.3. In-Tube SPME-MS/MS Optimization

The use of different ion transitions for each analyte favored detection without chromatographic separation (Table 2). Endogenous compounds interferers from plasma samples can suppress ionization of analytes (ESI), decreasing analytical sensitivity. Therefore, we directly coupled the monolithic capillary to the UV detector to optimize the time for analyte sorption and interferers exclusion. On the basis of Figure S1 (Supplementary Materials) and using acetonitrile as mobile phase, we found that approximately two minutes was sufficient to eliminate most plasma macromolecules. No analyte eluted between 2 and 10 min when we used acetonitrile as mobile phase. After 10 min, we changed the mobile phase from acetonitrile to water to elute the analytes from the monolithic capillary to the mass spectrometer (peak at approximately 13 min in Figure S1).

**Figure 4:**
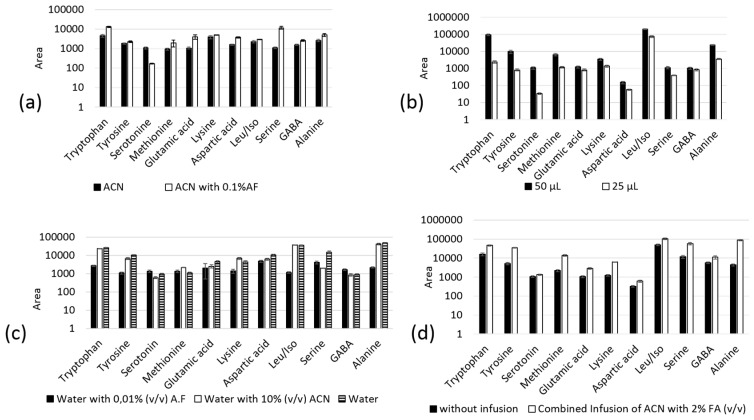
Effect of (**a**) sample solvent (acetonitrile and acetonitrile with 0.1% (*v*/*v*) formic acid on the pre-concentration step; (**b**) sample solvent volume (acetonitrile with 0.1% formic acid); (**c**) mobile phase for the elution step; and (**d**) post capillary infusion of acetonitrile with 2% formic acid (FA) on the performance of the in-tube SPME-MS/MS procedure.

In this work, sample solvent is defined as the solvent that was used to reconstitute the dried extract after protein precipitation. Figure 4 depicts the in-tube SPME-MS/MS optimization. Among the sample solutions evaluated during the pre-concentration step, 50 µL of acetonitrile with 0.1% (*v*/*v*) formic acid provided the highest sorption capacity (Figure 4a,b). The aqueous solutions evaluated did not presented adequate sorption due to their hydrophilic nature. 

The nature of the mobile phase used to elute the analytes affects the sensitivity of the method (Figure 4c). We selected the mobile phase on the basis of desorption of the analytes from the monolithic capillary and of ESI ionization. Formic acid addition to water improved ionization of the analytes ionization, whereas acetonitrile addition improved the analytical signal due to an increase in desolvation capacity. On the basis of Figure 4c, we selected water as mobile phase. Although formic acid and acetonitrile improve the ESI ionization, the presence of these additives in mobile phase decrease the desorption capacity of the analytes. 

Post capillary infusion of acetonitrile with 2% formic acid boosted the desolvation capacity and ionization of the analytes, thereby increasing the response (Figure 4d). Acetonitrile infusion reduced the water (mobile phase) dielectric constant and weakened electrostatic interactions between the analytes. The monolithic capillary was reused over 40 times without significant extraction efficiency loss (CV lower than 15%). Table 3 illustrates the optimized in-tube SPME-MS/MS procedure.

### 2.4. Adsorption Experiments

**Figure 5:**
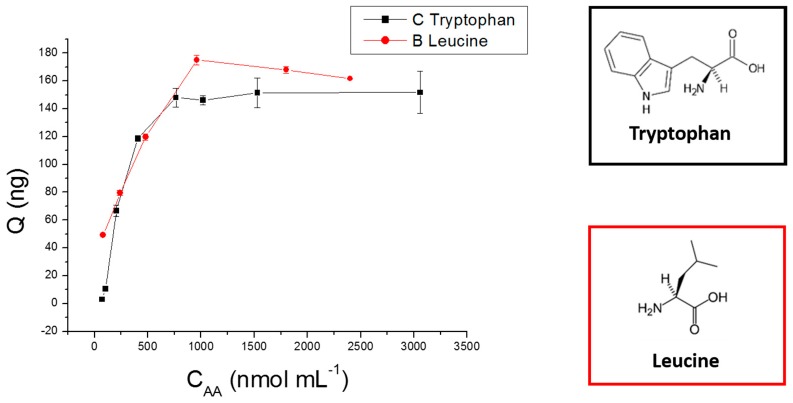
Sorption isotherm of the organic-inorganic hybrid silica-based monolithic capillary for tryptophan and leucine.

Figure 5 shows the sorption isotherms of tryptophan and leucine (representative analytes) and their respective structures. The monolithic capillary presented sorption capacity (binding affinity) of 6.53 µg cm^−3^ and 7.52 µg cm^−3^ for tryptophan and leucine, respectively. The sorption capacities determined for alanine, serine, glutamic acid, isoleucine, methionine, lysine, and aspartic acid were 5.73 µg cm^−3^, 7.44 µg cm^−3^, 2.86 µg cm^−3^, 5.35 µg cm^−3^, 2.56 µg cm^−3^, 5.54 µg cm^−3^, and 3.13 µg cm^−3^, respectively. Despite structural differences, these compounds have the same functional groups (hydroxyl, carboxyl, and amino), which are responsible for their sorption onto the monolithic capillary. Sorption isotherms for these AA are illustrated in the Supplementary Materials (Figure S2).

The structure of monolithic capillary is amorphous and homogeneous; the skeleton is porous and consists of interconnected particles (Section 2.2). Thus, the adsorption model closest to this application is the “external mass transfer model”, which describes mass transfer from de liquid phase (mobile phase) to the solid surface (internal surface of the capillary monolithic phase) into the monolithic capillary. Another model that could describe the sorption of amino acids onto the monolithic phase is the “pseudo first-order model”. However, in this work, the sorption of the target analytes onto monolithic capillary is reversible [51,52,53].

### 2.5. In-Tube SPME-MS/MS Analytical Validation

Analytical validation of the in-tube SPME-MS/MS method was based on the current FDA (Food and Drug Administration), and EMA (European Medicines Agency) international guidelines for the validation of a bioanalytical method [54,55,56]. 

According to the literature, the amino acids in plasma samples are stable for at least 24 h when stored at ambient temperature, over three freeze–thaw cycles, or when stored at −20 °C for six months [57,58]. After pre-treatment (Section 3.3), amino acids were stable for at least 24 h without a decrease in the area obtained in the in-tube SPME-MS/MS method for the sample stored at 10 °C.

Table 4 lists the linear range of the alanine, serine, isoleucine, leucine, aspartic acid, glutamic acid, lysine, methionine, tyrosine, and tryptophan evaluated in plasma samples spiked with standard solutions at different concentrations. Based on the analytical validation, the linear ranges of GABA and serotonin were not adequate. However, the p values of the lack of fit statistical test were higher than 0.05, which confirmed a good fit for all the other analytes. Therefore, the equation obtained during analytical validation can be used to quantify the target analytes [59,60]. 

The intra- and inter-assay precision presented CV values ranging from 1.6% to 14.0%. The intra- and inter-assay accuracy presented RSE values spanning from −11.0% to 13.8%, except for the values of lower limit of quantification (LLOQ) concentration, which ranged from −19.2 to 18.0. 

The matrix effect was evaluated by comparing the slopes of the calibration curves constructed for the analytes in plasma and aqueous solutions to evaluate parallelism between these analytical curves [55]. The Student’s *t*-test did not reveal any significant difference (*p* > 0.05) between these slopes, confirming parallelism and demonstrating that the matrix effect was not significant, Table 4. 

**Figure 6:**
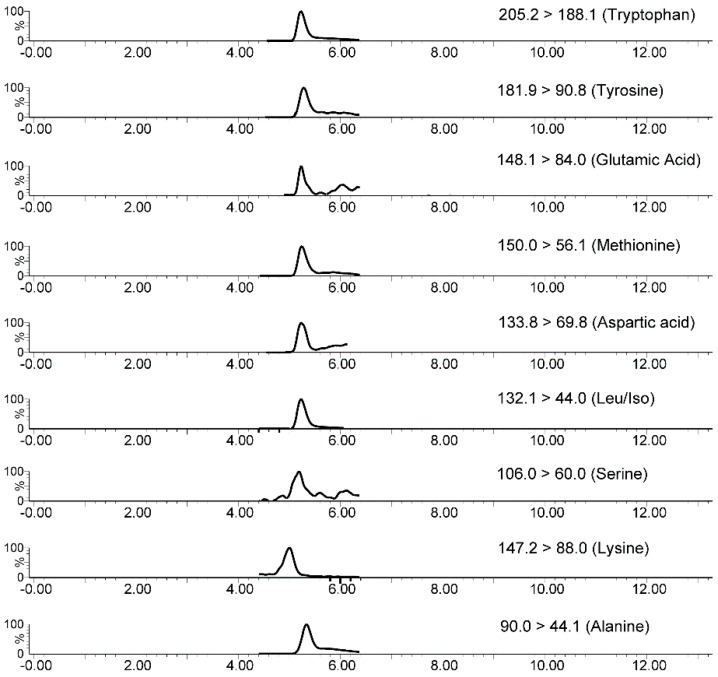
Representative in-tube SPME-MS/MS (SRM mode) chromatogram of plasma sample from a schizophrenic patient. Plasma concentrations: alanine = 243.7 nmol mL^−1^; serine = 155.1 nmol mL^−1^; leu/Iso = 199.9 nmol mL^−1^; aspartic acid = 13.5 nmol mL^−1^; methionine = 9.6 nmol mL^−1^; tyrosine = 35.9 nmol mL^−1^; tryptophan = 32.1 nmol mL^−1^; glutamic acid = 30.9 nmol mL^−1^; and lysine = 21.7 nmol mL^−1^.

The applicability of the proposed method was evaluated by analyzing plasma samples from six schizophrenic patients undergoing treatment with antipsychotics. Table 5 illustrates the average of these plasma concentrations and the standard deviations. Figure 6 shows a representative in-tube SPME-MS/MS (SRM mode) chromatogram of plasma sample from schizophrenic patient. The AA and NT concentrations determined in plasma from schizophrenic patients agreed with previously published data [9,61].

### 2.6. Comparison of the Proposed Method with Literature Methods

We compared the in-tube SPME-MS/MS method with other literature methods for AA and NT determination in plasma samples (see Table 6) [9,62,63,64].

Compared to recent protocols described in the literature, the in-tube SPME-MS/MS method offered the following advantages: online sample processing, high throughput analysis, and minimal organic solvent consumption (flow at 100 µL min^−1^) without addition of buffer solution to the mobile phase. The selectivity of both the hybrid silica monolithic capillary and the MS/MS system allowed direct coupling of the in-tube SPME technique to MS/MS. The proposed method did not present the lowest LLOQ values, but the obtained LLOQ values were adequate for the determination of the target analytes in plasma samples. In addition, the other analytical parameters (accuracy and precision) of the in-tube SPME-MS/MS method agree with the values established by the FDA and EMA guidelines.

## 3. Materials and Methods

### 3.1. Standards and Reagents

Alanine, serine, isoleucine, leucine, aspartic acid, glutamic acid, lysine, methionine, tyrosine, γ-aminobutiric acid (GABA) and tryptophan standards were purchased from SIGMA Sigma–Aldrich (St. Louis, MO, USA). Acetonitrile (UHPLC grade) was obtained from Sigma–Aldrich (St. Louis, MO, USA). The water used to prepare the solutions had been purified in a Milli-Q system (Millipore, Brazil). Tetraethylorthosilicate (TEOS, 98%), 3 cyanopropyltriethoxysilane (CN-TEOS, 98%), (3-aminopropyl) triethoxysilane (APTES, 98%), and cetyltrimethylammonium bromide (CTAB, 95%) were acquired from Aldrich (São Paulo, SP, Brazil).

### 3.2. Synthesis of Hybrid Silica-Based Monolithic Capillaries Bearing Amino and Cyano Groups

Organic-silica hybrid monolithic capillaries were synthesized by the sol–gel procedure in one step [39] with some modifications. Initially, the capillary was rinsed with 0.2 mol L^−1^ HCl solution for 30 min and then with water until the pH value of the outlet solution was 7.0. Subsequently, the capillary was flushed with 1 mol L^−1^ NaOH for 2 h, and then with water and methanol for 30 min. Finally, the capillary was purged with nitrogen at 160 °C for 3 h prior to use. After the capillary was pretreated, 5 mg of CTAB was mixed with water/ethanol solution (20 µL/100 µL) in a 1.5 mL Eppendorf vial. Next, 56 µL of TEOS, 28 µL of APTES, 28 µL of CN-TEOS, and 10 µL of ammonium fluoride aqueous solution were added to the initial solution, which was thoroughly vortexed at room temperature for 30 s. The pre-condensation mixture was quickly introduced with a syringe into the pretreated capillary of appropriate length. The capillary ends were sealed with two pieces of rubber and reacted at 40 °C for 15 h. The hybrid gel that emerged within the capillary was rinsed with ethanol to remove CTAB and synthesis residues, washed with water, and dried at 60 °C for 48 h. Different molar ratios of the precursors (TEOS, CN-TEOS, and APTES), aging temperatures (22 °C and 60 °C), CTAB amounts (5 and 7 mg), and ethanol/water ratios (100:20 and 50:50 *v*/*v*) were evaluated to optimize the synthesis procedure. Table 1 illustrates the experimental parameters that were assessed in triplicate assays.

### 3.3. Hybrid Silica Monolithic Capillary Characterization

The monolithic phase had its morphological and structural aspects evaluated by scanning electron microscopy (SEM). To this end, samples were coated with carbon in a Bal-Tec SCD050 Sputter coater instrument (FürstentumLiechtenstein, Cambridge, UK) for 120 s and analyzed under a Zeiss EVO50 scanning electron microscope (Cambridge, UK). Fourier transform infrared spectroscopy (FTIR) was conducted on a Shimadzu-IR Prestige-21 spectrometer (Barueri, Brazil), in KBr pellets, to identify chemical groups. Nitrogen sorption experiments were carried out at 77 K in a Micrometrics ASAP 2020 plus nitrogen sorption porosimeter (São Paulo, Brazil). Specific surface areas were determined by the Brunauer–Emmett–Teller (BET) method. X-ray diffraction (XRD) analyses were accomplished on a Siemens-Bruker D5005-AXS diffractometer (São Paulo, Brazil), with CuKa radiation, graphite monochromator, at λ = 1.5418 Å and 0.02° s^−1^, in the 5–70° (2θ) range.

### 3.4. Plasma Samples

Plasma samples were supplied by the Psychiatric Nursing staff of Hospital das Clínicas de Ribeirão Preto, University of São Paulo, Brazil. The plasma samples were collected in agreement with the criteria established by the Ethics Committee of Faculdade de Medicina de Ribeirão Preto, University of São Paulo, Brazil. Blood was collected by venipuncture and placed in tubes containing anticoagulants, EDTA. It was centrifuged immediately after collection, and plasma was stored at −80 °C.

Plasma proteins (200 µL) were precipitated with acetonitrile at a 1:2 (*v*/*v*, respectively) ratio. After vortex mixing for 1 min, the mixture was centrifuged at 9000 g (rpm) for 30 min. The supernatant (700 µL) was dried in a vacuum concentrator (Eppendorf, Brazil), and the dried extract was reconstituted with 50 µL of acetonitrile containing 0.1% formic acid (*v*/*v*) for the in-tube SPME-MS/MS procedure.

### 3.5. MS/MS Conditions

In-tube SPME assays were performed in a Waters ACQUITYUPLC H-Class system coupled to the Xevo^®^TQ-D tandem quadrupole (Waters Corporation, Milford, MA, USA) mass spectrometer equipped with a Z-spray source (electro spray ionization, ESI) operating in the positive mode. Selected reaction monitoring (SRM) transitions and optimal collision energies were optimized for each analyte (Table 2). MS parameters and source were optimized as capillary voltage of 0.50 kV, source temperature of 150 °C, desolvation temperature of 350 °C, desolvation gas flow of 600 L h^−1^ (N_2_, 99.9% purity), and cone gas flow of 20 L h^−1^ (N_2_, 99.9% purity). Argon (99.9999% purity) was used as collision gas. The dwell time was established for each transition separately, and the inter-scan delay was set at the automatic mode. Data were acquired by using the MassLynx V4.1 software (Waters Corporation, Milford, MA, USA).

### 3.6. In-Tube SPME Procedure

To optimize the in-tube SPME-MS/MS procedure, 300 µL of plasma sample was used. Parameters were optimized not only to enrich the monolithic capillary with the analytes, but also to improve MS/MS sensitivity. 

Different sample solutions (aqueous solution at different pH values (pH 4, 7, and 10), acetonitrile, and acetonitrile with 0.1% (*v*/*v*) formic acid), sample solution volumes (25 and 50 µL), and mobile phases to elute the analytes [water, water with 0.01% (*v*/*v*) formic acid, and water with 10% (*v*/*v*) acetonitrile] were evaluated.

The in-tube SPME-MS/MS system configuration was based on monolithic capillary (10 cm × 530 μm) and not analytical liquid column coupling to the MS/MS valve. 

**Figure 7:**
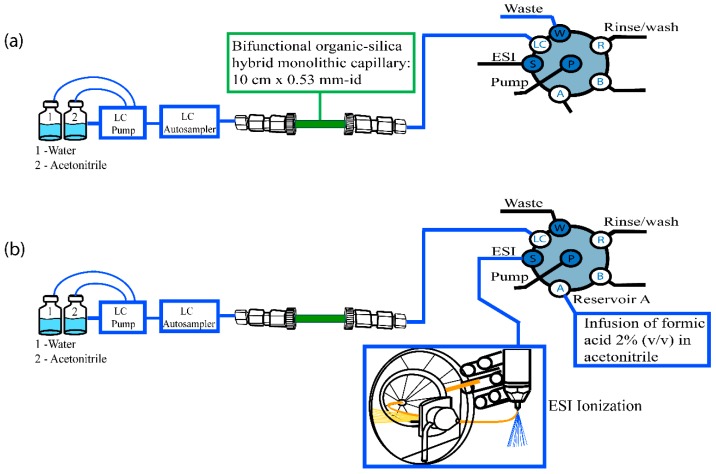
Scheme of in-tube SPME-MS/MS procedure). In the first step (**a**) (MS/MS valve in position 1), diluted sample (10 µL) was percolated through the monolithic capillary to pre-concentrate the analytes. After, (**b**) the valve was switched to position 2 for elution of the analytes From 4 to 7 min, acetonitrile with 2% (*v*/*v*) of formic acid was post-capillary infused.

The in-tube SPME procedure comprised three steps (Table 3). In the first step (MS/MS valve in position 1), diluted sample (10 µL) was percolated through the monolithic capillary to pre-concentrate the analytes and to exclude endogenous interferers; acetonitrile was used as mobile phase at a flow rate of 100 µL min^−1^ for 2 min. From 2 to 4 min, the valve was switched to position 2, and the analytes were eluted from the monolithic capillary to the mass spectrometer in tandem by using water as mobile phase at a flow rate of 100 µL min^−1^. From 4 to 7 min, acetonitrile with 2% (*v*/*v*) of formic acid was post-capillary infused to increase MS/MS sensitivity. From 7 to 12 min (third step), the valve was switched back to position 1, and the monolithic capillary was cleaned up with water and acetonitrile with gradient elution from 100% water to 100% acetonitrile at a flow rate of 100 µL min^−1^. Figure 7 illustrates a schematic diagram of in-tube SPME-MS/MS system configuration. 

### 3.7. Adsorption Capacity

Adsorption capacity was evaluated according to reported procedures [55,65]. Considering the target AA and NT chemical structures (cyclic and acyclic), tryptophan and leucine were selected as representative analytes. Standard tryptophan and leucine solutions were prepared in water at different concentrations (CAA, from 0.150 to 3.6 µmol mL^−1^) to evaluate the monolithic capillary maximum sorption capacity (Qmax ng cm^−3^). These solutions were injected separately into the in-tube SPME-MS/MS system; conditions described in Section 2.6 were employed. Qmax was estimated on the basis of the following equation Qmax = Q/Vm, where Q (ng) is the amount of analyte adsorbed onto the monolithic capillary as determined by calibration curves, and Vm = 22.6 cm^−3^ is the estimated monolithic phase volume immobilized into the capillary. Vm was calculated by using the capillary length (L) and internal radius (r) according to the following equation Vm = π r^2^ L, where π = 3.14, r = 265 µm, and L = 10 cm. Qmax was based on the saturation point of the plot of Q (ng) versus CAA.

### 3.8. Analytical Validation 

The linear ranges of the calibration curves were established in agreement with the AA and NT concentrations in plasma samples from schizophrenic patients [9,66]. Calibration curves were constructed by the standard addition method (standard solutions were added directly to the plasma samples): relative peak areas (analyte-to-IS) were plotted as a function of analytes concentration spiked in matrix samples in different ranges.

Accuracy and precision assays were carried out with plasma samples (representative amount of the plasma pool from different voluntaries) spiked with the analytes at five different concentrations, namely calibration controls (QC), LLOQ, low (QC), medium QC, high QC, and upper limit of quantitation (ULOQ). Precision assays were evaluated on the same day (intra-assays precision) and on three consecutive days (inter-assays precision) based on the coefficient of variation (CV) values; i.e., within 20%. Intra- and inter-assays accuracy were based on relative standard error (RSE%). RSE values should be within 15% of nominal values for QC samples.

Methionine-d3 and alanine ^13^C_3_^15^N were used as internal standards at concentrations of 30 and 100.0 nmol mL^−1^, respectively. Leucine and isoleucine present the same transitions because they have the same molecular weights and similar MS/MS characteristics [67]. Thus, these AA could not be separately determined in the triple quadrupole analyzer. These analytes were quantified on the basis of total concentrations; i.e., as a sum of the peak areas of the individual analytes.

The lower limit of quantification (LLOQ) is the lowest analyte concentration in a sample that can be reliably quantified with acceptable accuracy and precision. LLOQ is also the lowest concentration in the calibration curve. 

Carryover was assessed by injecting blank aqueous sample after a ULOQ concentration in plasma sample. Carryover in the blank aqueous sample should not be greater than 20% of the LLOQ or 5% for the internal standard.

The matrix effect was examined by comparing the average value of slopes of calibration curves obtained with four aqueous samples spiked with the target analytes at different concentrations with slopes of calibration curves obtained with four plasma samples from different patients [56]. Student’s *t*-test (*p*-value at a significance level of 0.05) was applied (Table S2 Supplementary Materials). 

## 4. Conclusions

Optimization of the synthesis procedure (molar ratios of the alkoxysilane precursors, aging temperatures, supramolecular template amounts, and ethanol/water ratios) and the use of ammonium fluoride as catalyst allowed us to develop an innovative organic-inorganic hybrid silica-based monolithic capillary with two bifunctional groups (cyano and amino). This reproducible capillary presented adequate sorption capacity, satisfactory permeability (low pressure), and high mechanical strength, which enabled it to be re-used over forty times without significant changes in extraction reproducibility or system pressure. 

Concerning morphology, the hybrid monolithic capillary tightly attached to the capillary inner wall and exhibited a homogeneous, continuous, and porous skeleton. FTIR analyses prove that the cyano and amino groups were incorporated into the monolithic capillary. 

The selectivity of the monolithic capillary (amino and cyano groups) and the MS/MS system allowed for direct coupling of in-tube SPME to the MS/MS system without the need for chromatographic separation. In agreement with the analytical validation assays, this automated innovative method is appropriate to determine the target AA and NT in plasma samples from schizophrenic patients for clinical studies involving short analysis time.

## Figures and Tables

**Table 1:** Optimization of the synthesis parameters.

Procedure	TEOS (µL)	APTES (µL)	CN-TEOS (µL)	H_2_O/EtOH (µL)	TEOS/APTES/CN-TEOS (µL)	Aging Temperature (°C)
1	56	28	28	100:20	2:1:1	60
2	38	38	38	100:20	1:1:1	60
3	56	56	0	100:20	1:1:0	60
4	56	0	56	100:20	1:0:1	60
5	56	28	28	50:50	2:1:1	60
6	56	28	28	50:50	2:1:1	22

**Table 2:** MS/MS (SRM) ion transitions, cone energy (DP), and collision energy (CE) for each analyte.

Analyte	Precursor Ion	Product Ion (Quantification)	DP (V)	CE (V)	Product Ion (Identification)
Tryptophan	205.2	146.0	20	12	188.1
Methionine	150.0	56.0	20	15	104.0
Methionine d3	153.1	56.0	20	15	107.1
Tyrosine	182.1	136.1	25	15	90.8
Leucine/Isoleucine	132.1	86.0	20	10	44.0
GABA	104.1	87.0	30	15	45.0
Serotonin	177.1	115.1	20	36	104.9
Glutamic acid	148.1	84.0	25	15	102.1
Lysine	147.2	88.0	25	15	107.0
Aspartic acid	134.1	74.0	20	12	88.0
Serine	106.0	60.0	20	10	88.0
Alanine	90.0	44.0	20	10	62.0
Alanine ^13^C_3_^15^N	94.2	47.1	20	10	64.8

DP = declustering potential; CE = collision energy.

**Table 3:** In-tube SPME-MS/MS procedure.

MOBILE PHASE A: Water B: Acetonitrile				
Time (min)	% A	% B	Valve Position	Comments
0.0	0	100	1	pre-concentration of analytes and exclusion of plasma macromolecules
2.0	100	0	2	Elution of analytes from monolithic capillary to mass spectrometer
4.0	100	0	2	Post capillary infusion of acetonitrile with 0.1% formic acid
7.0	100	0	1	Final elution step and start of gradient elution to clean up the capillary column

**Table 4:** Analytical curves data, Student’s *t*-test, and lack-of-fit statistical test of the in-tube SPME-MS/MS method to determine AA and NT in plasma samples.

Validation Parameters	Ala	Leu/Iso	Met	Ty	Try	Ser	Glu	Lys	Asp
Linearity (R^2^)	0.995	0.993	0.998	0.995	0.997	0.990	0.996	0.991	0.993
Slope Intercept	0.0013 0.4277	0.0110 2.4736	0.0704 0.6808	0.0057 0.1844	0.0251 0.6792	0.0024 0.3963	0.0022 0.0746	0.0046 0.0859	0.0057 0.0092
LOF (*p*-value)	0.997	0.803	0.489	0.251	0.876	0.892	0.972	0.251	0.875
Linear range (nmol mL^−1^)	45–360	15–300	12–102	10–102	9–96	12–210	12–90	12–102	6–36
Student’s *t*-test (*p*-value)	0.520	0.087	0.125	0.907	0.079	0.077	0.219	0.280	0.244

Ala = Alanine; Leu = Leucine; Iso = Isoleucine; Met = Methionine; Ty = Tyrosine; Try = Tryptophan; Ser = Serine; Glu = Glutamic acid; Lys = Lysine; Asp = Aspartic acid; LOF = lack of fit statistical test, (*p*-value at a significance level of 0.05).

**Table 5:** Average values of the AA and NT plasma concentrations (with standard deviation) determined in plasma samples from six schizophrenic patients (n = 6).

Plasma Concentration (nmol mL^−1^)	Ala	Leu/Iso	Met	Ty	Try	Ser	Glu	Lys	Asp
Average values	270.8 ± 60.1	246.1 ± 28.0	18.3 ± 5.1	40.5 ± 10.4	37.1 ± 9.7	143.0 ± 48.6	31.8 ± 11.2	20.6 ± 4.0	11.2 ± 7.5

Ala = Alanine; Leu = Leucine; Iso = Isoleucine; Met = Methionine; Ty = Tyrosine; Try = Tryptophan; Ser = Serine; Glu = Glutamic acid; Lys = Lysine; Asp = Aspartic acid.

**Table 6:** Comparison of the developed method with other methods described in the literature.

Analytes	Sample Preparation	Sample Volume (µL)	Analytical Method	Elution of Analytes (min)	validation Parameters (Intra and Inter Assays)	Ref.
10 amino acids	Protein precipitation	Plasma 50	UHPLC-MS/MS-Ascentis^®^ Express HILIC column (4.6 × 100 mm, 2.7 µm). MP: A = Ammonium acetate solution 10 mM; B = acetonitrile with 0.1% FA	3.2	LLOQ: 9.7–13.3 nmol mL^−1^ Precision: 2–10% (CV) Accuracy: −2.1–9.9% (RSE)	[9]
33 Amino acids	Protein precipitation	Plasma 100	Two columns: 1 - PGC column (Thermo Fisher Scientific, 3 µm Hypercarb, 4.6 mm i.d. × 50 mm), and 2 - fused-core column (Advanced Materials Technology, 2.7 µm Halo C18, 2.1 mm i.d. × 100 mm)	9.4	LLOQ: 0.1–10.0 nmol mL^−1^ Precision: 1.2–9.2% (CV) Accuracy: N.A	[63]
20 amino acids	Protein precipitation	Serum 100	UHPLC-MS/MS CROWNPAK CR-I(+) column (3.0-mm i.d. × 150 mm, 5 μm)	10.1	LLOQ: 0.1–10.0 nmol mL^−1^ Precision: 2.6–10.1% (CV) Accuracy: −12.8–12.4% (RSE)	[62]
22 amino acids	Protein precipitation	Plasma 10	HPLC-MS/MS Two Agilent Zorbax SB-C18 columns, (2.1 mm × 50 mm, 1.8 µm)	35.0	LLOQ: 0.01–0.07 nmol mL^−1^ Precision: 1.0–15.0% (CV) Accuracy: −12.8–12.4%	[64]
10 amino acids	In-tube SPME	Plasma 200	In-tube SPME-MS/MS with post capillary infusion	5.2	LLOQ: 6–45 nmol mL^−1^ Precision: 1.1–19.0% (CV) Accuracy: −14.4–19.6% (RSE)	This work

LLOQ = lower limit of quantification; CV = coefficient of variation RSE = relative standard error. N.A = not avaliable.

## Supplementary Materials

The supplementary materials are available online.
