# Supplementary material for: A Dual Ligand Sol–Gel Organic-Silica Hybrid Monolithic Capillary for In-Tube SPME-MS/MS to Determine Amino Acids in Plasma Samples
Source: Molecules. 2019 Apr 27;24(9):1658. doi: 10.3390/molecules24091658 (PMC6540176; doi:10.3390/molecules24091658)
Supplement: Supplementary file 1 [file molecules-24-01658-s001.pdf]

# Supplementary material

Tables Caption

Table S1. Precision and accuracy of in-tube SPME-MS/MS method

| | Concentration (nmol $mL^{-1}$) | Precision Intra (RSD/%) | Precision Inter (RSD/%) | accuracy intra (%) | Accuracy inter (%) |
|---|---|---|---|---|---|
| Try | 9[a] | 3,5 | 8,4 | 9,1 | 16,4 |
| | 15[b] | 2,9 | 6,7 | -10,6 | -8,3 |
| | 48[c] | 4,6 | 4,6 | -4,9 | 10,4 |
| | 72[d] | 5,35 | 7,71 | -6,8 | 8,2 |
| | 96[e] | 4,62 | 5,39 | 2,7 | 9,7 |
| Ty | 6[a] | 9,9 | 7,1 | -12,3 | -15,6 |
| | 10[b] | 11,4 | 9,3 | 10,3 | -11,5 |
| | 48[c] | 2,6 | 2,7 | -8,0 | 9,8 |
| | 75[d] | 7,1 | 3,9 | 9,7 | 8,7 |
| | 102[e] | 10,5 | 7,4 | -10,6 | -11,9 |
| Met | 12[a] | 5,9 | 2,6 | 3,8 | 11,1 |
| | 18[b] | 9,6 | 6,8 | -2,7 | -4,8 |
| | 48[c] | 5,0 | 7,8 | -5,6 | -8,3 |
| | 75[d] | 7,2 | 6,3 | 4,38 | -7,7 |
| | 102[e] | 2,8 | 2,6 | 0,14 | 0,32 |
| Glu | 12[a] | 10,8 | 8,8 | -16,0 | -14,7 |
| | 18[b] | 5,9 | 2,9 | 11,3 | 10,1 |
| | 48[c] | 5,3 | 1,5 | -6,0 | -8,2 |
| | 75[d] | 2,5 | 1,8 | 4,7 | 3,1 |
| | 90[e] | 4,6 | 2,3 | 0,63 | 2,0 |
| Lys | 12[a] | 18,1 | 19,0 | 17,9 | 19,6 |
| | 24[b] | 10,3 | 13,2 | -8,0 | -9,4 |
| | 42[c] | 9,6 | 8,2 | -6,1 | -7,5 |
| | 81[d] | 10,0 | 11,7 | 1,3 | 2,33 |
| | 102[e] | 8,4 | 7,8 | 6,1 | 5,0 |
| Asp | 6[a] | 8,1 | 10,8 | -11,1 | 15,2 |
| | 9[b] | 5,5 | 7,9 | 6,01 | -9,5 |
| | 18[c] | 4,5 | 7,3 | -6,11 | -7,0 |
| | 30[d] | 10,1 | 12,5 | 0,2 | 2,3 |
| | 36[e] | 14,3 | 12,6 | 1,4 | 7,3 |
| Leu/Iso | 15[a] | 3,1 | 3,4 | 18,3 | 19,6 |
| | 45[b] | 6,0 | 7,6 | 12,6 | 10,3 |
| | 180[c] | 1,4 | 3,2 | 8,7 | 11,7 |
| | 210[d] | 3,7 | 12,4 | 10,0 | 9,6 |
| | 300[3] | 1,1 | 4,4 | 9,2 | 8,0 |
| Ser | 12[a] | 17,4 | 18,8 | 18,7 | 19,3 |
| | 30[b] | 10,8 | 9,1 | -14,4 | -13,7 |
| | 120[c] | 11,6 | 10,0 | -12,0 | -13,9 |
| | 150[d] | 8,8 | 5,2 | 14,1 | 12,9 |
| | 210[e] | 7,1 | 9,0 | 13,5 | 14,2 |
| Ala | 45[a] | 6,5 | 11,5 | 6,9 | 8,0 |
| | 90[b] | 8,8 | 12,6 | 5,2 | 2,8 |

| | | | | |
|---|---|---|---|---|
| 180[c] | 12,1 | 14,0 | 8,6 | 4,9 |
| 300[d] | 8,6 | 14,9 | 7,9 | 7,7 |
| 360[e] | 5,16 | 8,8 | 7,0 | 3,1 |

Ala = Alanine; Leu = Leucine; Iso = Isoleucine; Met = Methionine; Ty = Tyrosine; Try = Tryptophan; Ser = Serine; Glu = Glutamic acid; Lys = Lysine; Asp = Aspartic acid. The superscript letter a, b, c, d, and e correspond to LOQ, low QC, medium QC, high QC, and ULOQ concentrations respectively.

Table S2. Student's t-test data

| analyte | Slope (plasma) | Slope (water) | Student's t-test (p-value) |
|---|---|---|---|
| Tryptophan | 0.0269 | 0.0281 | |
| | 0.0231 | 0.0312 | 0.094 |
| | 0.0295 | 0.0331 | |
| | 0.0251 | 0.0271 | |
| Tyrosine | 0.0058 | 0.0066 | |
| | 0.005 | 0.0057 | 0.810 |
| | 0.0059 | 0.0059 | |
| | 0.0056 | 0.0054 | |
| Methionine | 0.0675 | 0.0672 | |
| | 0.0661 | 0.0704 | 0.204 |
| | 0.0687 | 0.0694 | |
| | 0.072 | 0.0734 | |
| Glutamic acid | 0.0025 | 0.0022 | |
| | 0.002 | 0.0028 | 0.248 |
| | 0.0018 | 0.0027 | |
| | 0.0023 | 0.0025 | |
| Aspartic acid | 0.0055 | 0.0063 | |
| | 0.0050 | 0.0048 | 0.343 |
| | 0.0045 | 0.0057 | |
| | 0.0061 | 0.0059 | |
| Leu/Iso | 0.0116 | 0.0109 | |
| | 0.0112 | 0.0145 | 0.395 |
| | 0.0111 | 0.0112 | |
| | 0.0126 | 0.0132 | |
| Lysine | 0.0045 | 0.0062 | |
| | 0.0055 | 0.0056 | 0.535 |
| | 0.0047 | 0.0059 | |
| | 0.0059 | 0.0047 | |
| Serine | 0.0023 | 0.0024 | |
| | 0.0025 | 0.0021 | 0.173 |
| | 0.003 | 0.0028 | |
| | 0.0029 | 0.0022 | |

| | | | |
|---|---|---|---|
| Alanine | 0.0015 | 0.0016 | 0.519 |
| | 0.0015 | 0.0013 | |
| | 0.0015 | 0.0016 | |
| | 0.0017 | 0.0014 | |

Figures Caption

Figure S1. Optimization of sorption of the analytes.

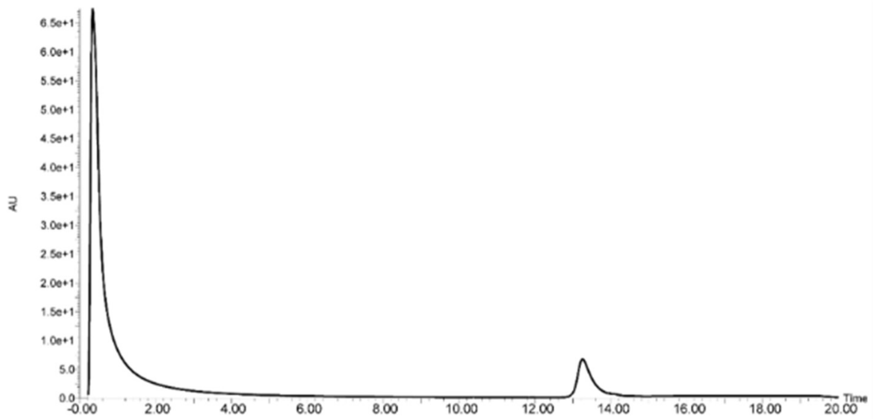


Figure S1. In-tube SPME-UV chromatogram of a volunteer's plasma sample after protein precipitation. Mobile phase: 100% acetonitrile from 0 to 10 min and 100% water from 10 to 20 min, $\lambda = 280$ nm.

Figure S2. Sorption capacities of target amino acids.

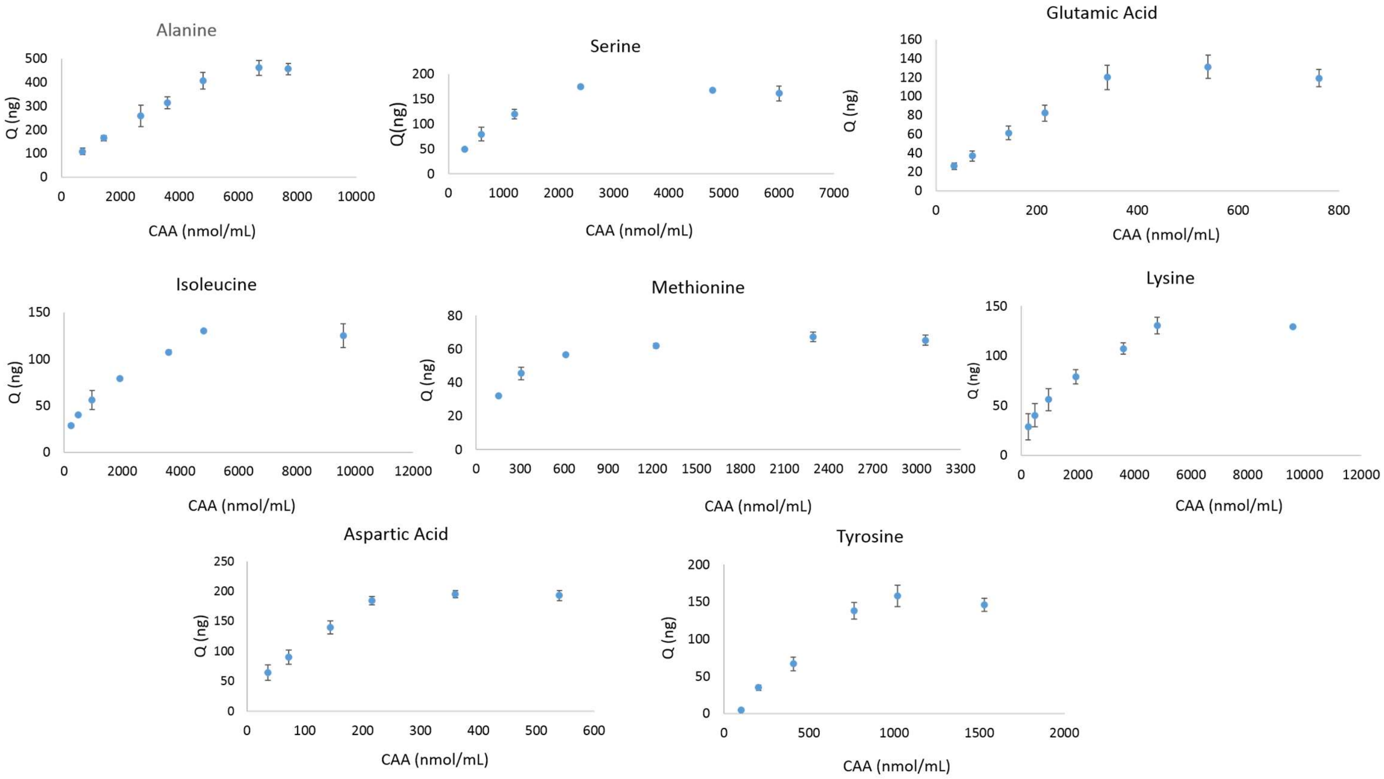


Figure S2. Sorption isotherm of the organic-inorganic hybrid silica-based monolithic capillary for alanine, serine, glutamic acid, isoleucine, methionine, lysine, and aspartic acid.
